# Preparation and Filling of Root Canals

**Published:** 1889-10

**Authors:** G. F. Root

**Affiliations:** Philadelphia


### PREPARATION AND FILLING OF ROOT CANALS.[1]

BY G. F. ROOT, D.D.S., PHILADELPHIA.

In presenting the subject I will commence with the devitalization and removal of a living pulp, as that is about the first step in the preparation of a root canal. There are several ways of obtaining the desired result, but I shall mention only two, as they are the principal methods now in use. I refer to escharotic agents and mechanical extirpation.

The latter is an operation that presents numerous difficulties and objections, owing principally to the reluctance of patients to stand the pain, and the inability in many cases of obtaining free access to the canals. To perform the operation, first obtain a free opening to the canal, and then, by passing a broach with a sharp barb quickly to the apex, between the pulp and tooth, rotate several times, whereupon the pulp can be withdrawn. It is an operation that should not be performed except in extreme cases, such as lack of time, or where we have a pulp more or less benumbed or deadened to pain. There is another way advocated by some operators, who claim almost no pain in its performance. This is done by sharpening a stick of wood to as near the size of the canal as can be judged, and, after dipping it in carbolic acid and having the pulp freely exposed, drive the peg of wood through the tissues of the pulp into the canal with a sharp blow of the mallet; and when the peg is withdrawn the pulp comes away with it, or, if it should not, it can easily be removed with a broach. I have never tried this method, and therefore cannot speak from experience; but should think, even leaving out of the question the fact of the fearful pain it must necessarily cause, the impossibility of forming the peg to the proper size, and the possibility of a crooked canal, would deter most careful operators from trying the experiment.

Operations should be performed with the very smallest amount of pain possible to the patient, and I therefore oppose anything like extirpation, except, as I have said before, in extreme cases. Most patients will willingly give time to save pain; and although the devitalization of a pulp is not performed with entire freedom from pain, yet there is a vast difference in the amount of pain endured

---

[1] Read at the twenty-first annual meeting of the Pennsylvania State Society, Cresson, Pennsylvania, July 30, 1889.

in performing the two operations. Arsenic is the escharotic agent generally used, in combination often with some other drug or chemical, to alleviate the pain usually caused. There are other escharotic agents that are sometimes used, but as arsenic is the quickest I will confine my remarks to that alone. I have tried several obtundents in connection with the arsenic, but have had more success with arsenic and sulphate of morphia (equal parts) than with any other preparation. I have used Dr. Kirk's preparation of cocaine in combination with the arsenic, but had comparatively little success with it, as it did not appear to alleviate the pain any more than morphia. However, there is nothing, so far as I know, that will give entire freedom from the pain caused by the arsenic.

It is important, before applying the drug, to reduce as far as possible the irritated condition usually found when first we see the case. One application usually is all that is required, but sometimes it is necessary to make several before devitalization is complete. I generally allow from twenty-four to seventy-two hours for the first application to take effect. If within the course of twenty-four hours the patient should have severe pain, lasting two or three hours, the pulp can be removed at the end of the first twenty-four hours, with but very little pain; but if there should be no complaint of severe toothache, it will usually take the full seventy-two hours for the devitalization to be complete, and frequently it will require one or more applications of arsenic. Of course, it is not absolutely necessary to have pain in a tooth before devitalization takes place, but in a large majority of cases you will find there has been more or less pain before the pulp dies. Very often the pulp will be only partially dead, sometimes in one of the roots of a tooth and not in others; or it may die only part way down the canal. In such cases I remove the dead portion, stop any hemorrhage that may occur, and reapply arsenic; and so on at each sitting until satisfied I have removed all the pulp. I have found Donaldson's nerve-broaches or bristles the best instruments to remove pulps by running them down the canal between the pulp and the tooth to the apex, the same as in extirpation, only we do not have to be so quick, and then by twisting several times the barbs will become entwined in the pulp-tissue and can readily be removed, unless, as we sometimes find, the pulp is very much softened and disintegrated, and we have to remove it piecemeal; in such cases it is all-important to remove all the pulp, or else we are sure to have trouble after the tooth is filled; in such cases, after all the pulp possible is removed with the bristles, I usually wipe the canal out several times with cotton entwined on a fine broach saturated in carbolic acid, care being taken not to force any *débris* through the apical foramen; the small particles that will not come away with the bristle will cling to the cotton and be withdrawn with it, thus leaving very little chance for any to remain in the canal. After we are satisfied we have a clean canal, and it is ready for filling, we are confronted with the question, Shall we fill at once? We will of course have to be governed very much by circumstances, but I believe that, with very few exceptions, it is far better to insert an antiseptic dressing in the canal, and leave for a day or two, than to fill immediately, for the reason that usually there is more or less hemorrhage which it is almost impossible to stop at once, and also that very often we have more or less irritation at the apex due to the use of the arsenic, thus lessening the chances for success. I usually use carbolic acid on cotton for the dressing, as it not only answers the purpose of an antiseptic, but through its coagulating power stops any hemorrhage that may occur. An application of iodine and tincture of aconite root, equal parts, over the gum, will usually stop any irritation at the end of the root.

We meet another class of teeth,—those in which the pulps are dead; and of these several different kinds, one in which the pulp has been dead for some time without giving any sign of trouble, another in which there is some congestion and irritation at the apex, and others in which we have an abscess to contend with; but the preparation of the canal in all of these cases amounts to the same thing, so I will consider them all under one method of treatment. After the canal has been freely exposed, if the pulp is not softened and broken up, it can easily be removed by using the same means previously spoken of, care being taken not to force any putrescent matter through the apex in inserting the instrument; but in case the pulp is softened and broken down, and we find nothing but pus and putrefied matter, I first take some absorbent cotton or bibulous paper on a broach and absorb all the fluid possible; then with a broach remove all the *débris*, using the same care as before in regard to forcing anything through the apex. I then insert a shred of cotton, saturated in carbolic acid, loosely in the canal, and seal with cotton alone, in order to give free outlet to any gases that may be formed; at the next sitting, after removing the dressing, I syringe well with tepid water, and, after drying well, inject peroxide of hydrogen freely into the canal with cotton on a broach; again dry and inject bichloride of mercury solution (one to five hundred); dry again and insert cotton saturated in carbolic acid, this latter to keep the canal sweet and clean until the next sitting. I thus treat the canal several times, until there are no signs of pus or any offensive odor, and then it is ready for filling.

The bichloride of mercury is the best antiseptic, and I believe it is the strongest known; it is to be used with care, owing to its being such a deadly poison. I believe I have had more success with this method than with any other I have used, and would strongly recommend those who have never used it to give it a good trial, and I feel sure it will give satisfaction.

Where I find a canal too small for a broach, I generally enlarge it with a canal-drill, commencing with a small size and then a larger one, and so on; considerable care is required to prevent drilling through the wall of the tooth, but only in such cases where there is a small canal do I think it at all necessary to use a drill.

When the canal is ready for filling, the question arises, What shall we fill with? Gold? No: as it is a waste of time and labor; other filling-materials answer the same purpose, and are easier of manipulation. Amalgam? No: as there is some liability of its discoloring the tooth. We now have left three materials that are almost in universal use,—oxychloride of zinc, gutta-percha, and plain cotton. There is no doubt but what the first-named would make by far the best material for the purpose, if it were not for there being a chance of future trouble at the end of the root, no matter how careful we are in our treatment. I do not believe we can be positive of success, for with all our care it is just possible that a small amount of decayed tissue may be left in the canal, and a very small particle would cause a mountain of trouble; therefore I do not think it advisable to fill with the cement, at least at first, owing to the extreme difficulty of removing if necessary. I would use either gutta-percha or the cotton for about one year, or as long as a good cement filling would last, and at the end of that time, if there was no sign of trouble, and on removing the filling I found no trace of putrescent matter, I might then fill with oxychloride with safety.

To fill with gutta-percha, I dissolve some of the material in chloroform to about the consistency of cream, and then with a broach work a little into the canal, and then take one of those points made for the purpose, and insert in the canal as far as the apex. If care is taken not to get too much of the liquid gutta-percha in the canal, and it is not too thin, there is very little danger of forcing any through the foramen; and even if a small portion should go through, I do not think it would cause any trouble. I have also had success in filling with cotton. I take a shred of cotton and roll tightly with my fingers; then, dipping it in carbolic acid, carry it to the apex with a smooth broach and pack in firmly; this makes about as reliable a filling as any; and although there are failures reported, I believe, if the truth be known, not more than with the other materials.

I am sorry that I cannot offer the profession some material that will combine the good qualities of all I have mentioned, leaving out the bad; but I hope that some member will conceive of something before long that will answer the same purpose.

